# Multivariate Mendelian randomization provides no evidence for causal associations among both psoriasis and psoriatic arthritis, and skin cancer

**DOI:** 10.3389/fimmu.2023.1252720

**Published:** 2023-09-19

**Authors:** Nianzhou Yu, Jiayi Wang, Yuancheng Liu, Yeye Guo

**Affiliations:** ^1^ Department of Dermatology, Hunan Key Laboratory of Skin Cancer and Psoriasis, Hunan Engineering Research Center of Skin Health and Disease, Xiangya Hospital, Central South University, Changsha, Hunan, China; ^2^ National Clinical Research Center for Geriatric Disorders, Xiangya Hospital, Central South University, Changsha, Hunan, China; ^3^ Xiangya School of Medicine, Central South University, Changsha, China

**Keywords:** psoriasis (PsO), psoriatic arthritis (PsA), skin cancer, multivariate Mendelian randomization (MVMR), effect

## Abstract

**Background:**

Some retrospective studies reported that psoriasis (PsO) and psoriatic arthritis (PsA) may have been associated with an elevated risk of skin cancer. The causal associations among them remain unclear.

**Objectives:**

To evaluate the causal association of among both PsO and PsA, and skin cancer.

**Methods:**

We performed large-scale two-sample and Multivariate Mendelian randomization analyses to examine whether there is a causal relationship between PsO and PsA, and skin cancer, encompassing basal cell carcinoma (BCC), cutaneous squamous cell carcinoma (cSCC), and cutaneous melanoma (CM).

**Results:**

Genetically predicted PsO, per log-odds ratio increase, showed no significant association with the risk of BCC, cSCC, and CM. The odds ratios (with corresponding 95% confidence intervals) for BCC, cSCC, and CM were 1.00 (0.99,1.01) (P_Ivw_ = 0.990), 0.94(0.89, 1.00) (P_Ivw_ = 0.065), and 0.99 (0.98, 1.01) (P_Ivw_ = 0.239), respectively. PsA showed a significant association with a decreased risk of BCC, with odds ratios (with corresponding 95% confidence intervals) of 1.00 (1.00, 1.00) (P_Ivw_ = 0.214) and 1.00 (1.00, 1.00) (P_Ivw_ = 0.477), respectively. Univariate analysis of the FinnGen database demonstrated PsA did exhibit a significant association with the decrease risk of BCC, with an odds ratio of 0.94(0.90,0.99) (P_Ivw_ = 0.016). However, this association disappeared after other risk factors were adjusted.

**Conclusions:**

Our findings suggest no causal association between PsO and PsA and the genetic risk of skin cancer. Further observational studies are required to elucidate the relationship among PsO, PsA, and skin cancer.

## Introduction

Psoriasis (PsO) is a relatively common chronic inflammatory skin condition. It may result from a combination of genetic predisposition and triggers such as infections, trauma, stress, and medications. Typical manifestations include itchy, scaly, pink patches, most commonly found on the elbows, knees, and scalp (1). Recent comprehensive systematic reviews have reported an increased propensity for skin cancer among individuals diagnosed with PsO (2–4). This association is believed to arise from prolonged skin inflammation and the use of certain medications to treat psoriasis, which can render the skin more susceptible to carcinogenic changes (5). Psoriatic arthritis (PsA) is a form of inflammatory arthritis impacting both the joints and entheses, which are the junctions where tendons and ligaments attach to bone. PsA is also considered to be associated with skin cancer. In a 2019 study that reviewed a total of 43,115 PsA patients across 9 cohort studies, it was found that the use of methotrexate, leflunomide, hydroxychloroquine, or sulfasalazine as treatments was associated with an increased risk of non-melanoma skin cancer (NMSC) (6).

While certain studies have observed an elevated risk of skin cancer associated with PsO and PsA, the precise underlying connection remains unclear, with some research even yielding difference findings. A comprehensive understanding of this causal association would facilitate a deeper understanding of the underlying mechanisms driving their respective risk profiles. Mendelian Randomization (MR) analysis is an epidemiological approach that enhances causal inference by using genetic variants as instrumental variables for exposures, with the advantage of being less likely to be affected by confounding factors than observational studies (7). Furthermore, MR can reduce reverse causality since Mendelian genotypes cannot change due to the onset and progression of diseases (7). Hence, our study aims to explore the causal association between PsO and PsA, and skin cancer through univariate and multivariate Mendelian randomization analyses (MVMR).

## Materials and methods

### Study design

**Figure 1:**
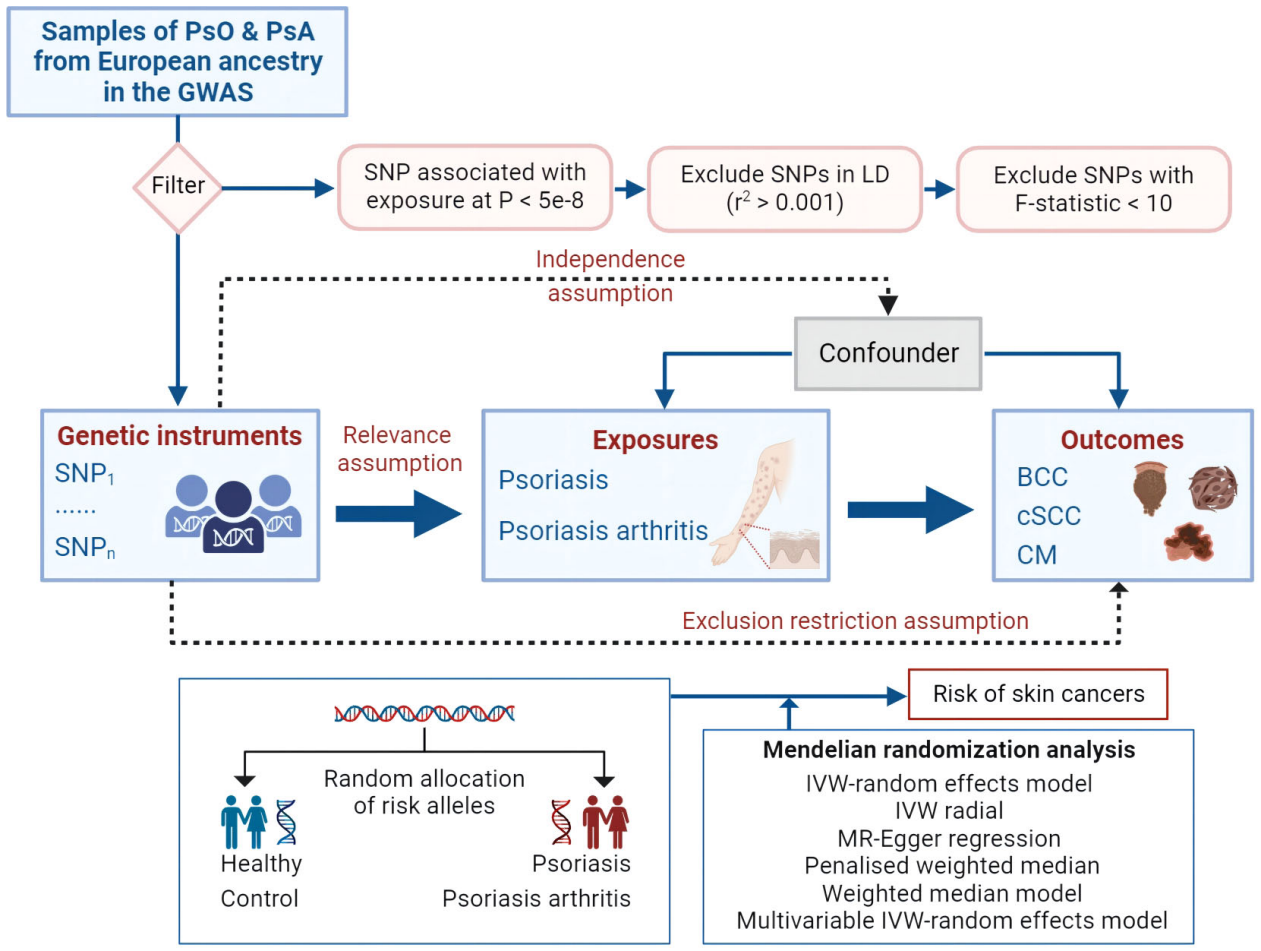
Mendelian randomization concept and assumptions. Schematic illustration depicting the causal relationship between both PsO and PsA and skin cancer through MR analyses. PsO, Psoriasis; PsA, Psoriatic Arthritis; BCC, Basal cell carcinoma; cSCC, Cutaneous squamous cell carcinoma; CM, Cutaneous Melanoma; MR, Mendelian randomization; SNPs, single nucleotide polymorphisms, LD, linkage disequilibrium; IVW, inverse variance weighted.

**Figure 1** outlines the study’s design and data sources in a schematic representation. Our investigation is underpinned by data pertaining to PsO, PsA and skin cancer meticulously sourced from the UK Biobank (8), the FinnGen study (9) and summary statistics from Genome-Wide Association Studies (GWASs) (**Table S1**). Our analytical approach commenced with an exploration of genetic correlations and MR associations between the genetic predisposition to PsO and PsA, and various subtypes of skin cancer. Subsequently, we conducted Multivariable MR Analysis (MVMR) to account for potential confounding factors related to skin cancer. All utilized data consist of publicly available GWAS summary statistics, thereby obviating the need for additional ethical approval or informed consent.

### Data sources

The single nucleotide polymorphisms (SNPs) strongly associated with PsO (P < 5 × 10^(-8)) were obtained from a genome-wide association meta-analysis, which included 10,588 PsO patients and 22,806 individuals of European ancestry as controls (10). It includes five GWAS datasets, which consist of the Psoriasis Collaborative Association Study, Kiel, Wellcome Trust Case-Control Consortium 2, Psoriasis Society Genetics Extension, and Genetic Analysis of the Psoriasis Consortium. For PsA, the latest FinnGen release 9 gene database was employed, encompassing 3,186 cases and 240,862 controls (9).

Skin cancers predominantly encompass basal cell carcinoma (BCC), cutaneous squamous cell carcinoma(cSCC), and cutaneous melanoma (CM). Each outcome trait corresponds to two sets of data obtained from different databases. The BCC dataset was sourced from a genome-wide association meta-analysis (11), encompassing 17,416 cases and 375,455 controls, with its primary source being the UK Biobank. Additionally, we incorporated data from FinnGen (18,982 cases and 305,750 controls). The cSCC data were obtained from Neale Lab (404 cases and 336,755 control) and FinnGen (3,251 cases and 287,157 controls). The data for CM was sourced from the UK Biobank and FinnGen, comprising a sample size of 3,751 cases and 372,016 controls, and 2993 cases and 290130 controls. This ensured that exposure and outcomes originated from distinct databases, thus avoiding population overlap. Furthermore, we obtained three exposure factors known to be associated with an elevated risk of BCC (12) from the GWAS: Ease of skin tanning (Dataset: ukb-b-533), radiation-related disorders of the skin and subcutaneous tissue (Dataset: finn-b-L12_RADIATIONRELATEDSKIN), and telomere length (Dataset: ieu-b-4879) (Summary in **Table S1**).

### Instrumental variables

To investigate potential causal links and associations between PsO and PsA and skin cancer, it is essential to select valid instrumental variables (IVs) that satisfy three key assumptions: (1) the correlation hypothesis, (2) the exclusivity hypothesis, and (3) the independence assumption (13). We selected SNPs as IVs up to the genome-wide significance threshold (P < 5 × 10−8). To ensure the independence of each SNP, we applied a linkage disequilibrium (LD) factor (r2) of 0.001 and a clumping window width of 10000 kb (14). Subsequently, we extracted information on SNPs from the database. We eliminated missing SNPs and set the minor allele frequency (MAF) at 0.01 (15). Additionally, we excluded all SNPs with palindromic structures to mitigate the influence of alleles on the results. To examine the presence of bias in the causal relationship between skin cancer and PsO and PsA due to weak IVs, we calculated the power using the F statistics [F = R2 × (N − 2)/(1 − R2)] for each SNP (16). When the F-statistic was less than 10, we considered the used SNP a weak IV and excluded it from the analysis (17). To evaluate the potential influence of confounding factors, we utilized the PhenoScanner V2 online tool (18). SNPs that showed associations with known confounders of PsO and PsA were subsequently excluded from the analysis.

### Mendelian randomization analysis

We utilized four different approaches, namely MR-Egger, weighted median, random-effect inverse variance weighted (IVW), and weighted mode, to perform the MR analysis and calculate causal estimates between PsO and PsA and the risk of skin cancer. Each approach has its specific requirements and assumptions (19). In the MR analysis conducted in this study, a causal relationship between skin cancer and the risk of PsO and PsA was considered when a significant p-value (p < 0.05) derived from any of these four methods was detected. By employing these approaches, the study aimed to assess the potential causal effects of skin cancer composition on PsO and PsA and provide insights into their relationship. Additionally, a reverse MR analysis was performed.

### Sensitivity analysis

The sensitivity analysis encompassed a heterogeneity test and a multiplicity of validity test. To confirm IV heterogeneity, Cochran’s Q-test was employed, and a p-value of less than 0.05 was considered indicative of the absence of heterogeneity (16). For evaluating the magnitude of horizontal pleiotropy, MR-PRESSO aggregated the residuals for each SNP. The MR-PRESSO outlier test facilitated the identification of outlier SNPs that contributed to pleiotropy at the overall level (20). The impact of individual outliers on the overall results was assessed using a leave-one-out analysis, calculating the remaining SNP effects after iteratively removing each SNP (21). Both MR-PRESSO and the leave-one-out analysis methods were employed to identify and eliminate SNPs exhibiting pleiotropy or heterogeneity (20). We applied MR Steiger filtering to determine the direction of causality for each instrumental variable on exposures and outcomes. The Steiger filtering method assumes that a valid instrumental variable should explain more variation in exposure than in outcome, with the direction of the instrument classified as “TRUE” if it meets the criteria and “FALSE” otherwise (22).

### Multivariate Mendelian randomization analysis

MVMR analyses based on the IVW method (which assumes all variants are ‘valid’ instrumental variables; that is, the SNP effect on BCC outcome is solely through its effect on the exposure/risk factor (23) were undertaken to determine genetic relationships between exposure variables and the association of these variables with outcomes.

### Statistical analysis

The MR analyses were conducted using the R (version 4.3.0) computational environment [https://www.R-project.org/], utilizing the “TwoSampleMR” (24),”MVMR” (25) and “MR-PRESSO” (20) packages. The R package ‘ forestploter’ was employed for generating certain figures. Statistical significance for causal effects was determined using a p-value threshold of less than 0.05.

## Results

**Figure 2:**
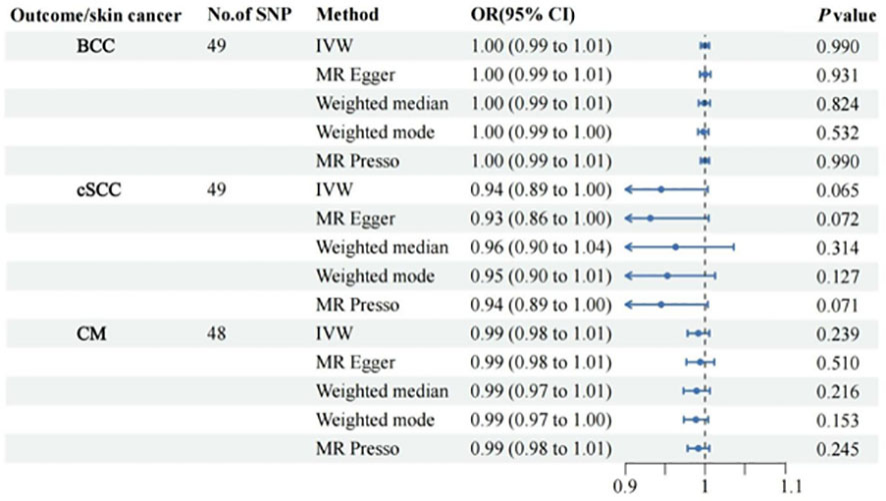
Forest plots for the associations of genetic susceptibility to BCC, cSCC and CM with different Mendelian randomizations of Psoriasis. BCC, Basal cell carcinoma; cSCC, Cutaneous squamous cell carcinoma; CM, Cutaneous Melanoma; OR, odds ratio; CI, confidence interval. Statistical significance: p < 0.05.

**Figure 3:**
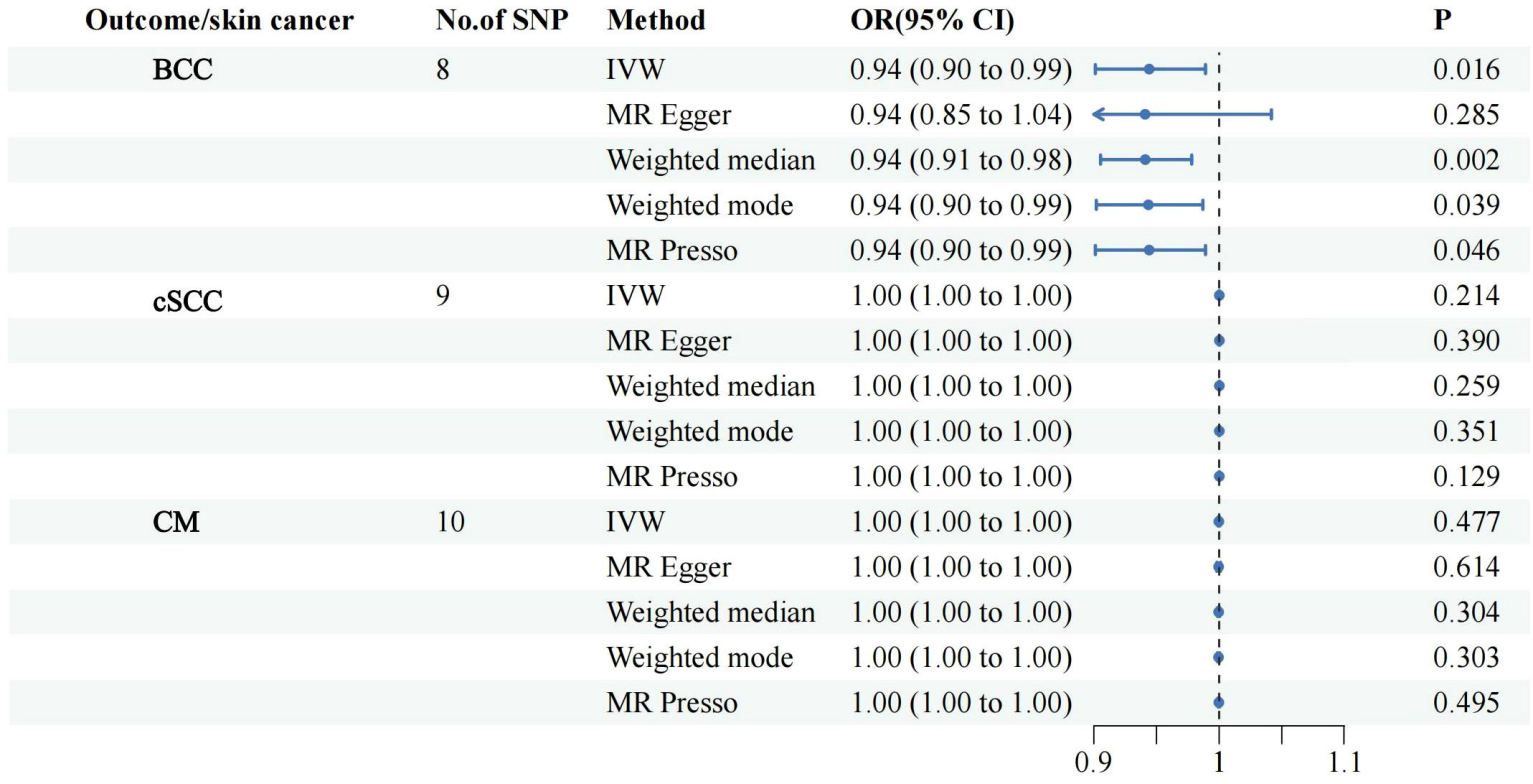
Forest plots for the associations of genetic susceptibility to BCC, cSCC and CM with different Mendelian randomizations of PsA. PsA, Psoriatic Arthritis; BCC, Basal cell carcinoma; cSCC, Cutaneous squamous cell carcinoma; CM, Cutaneous Melanoma; OR, odds ratio; CI, confidence interval. Statistical significance: p < 0.05.

**Figure 4:**
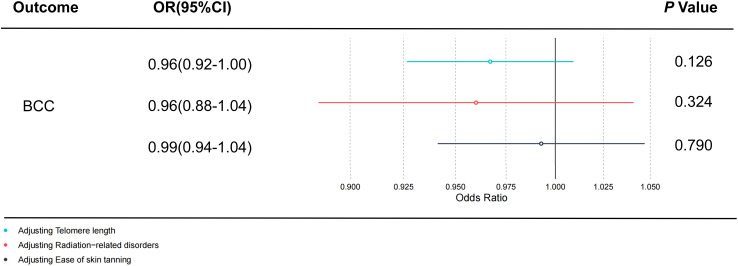
The direct causal effect of PsA on BCC by adjusting skin tanning, radiation-related disorders, and telomere length. The reported values were calculated by the random effects IVW method. PsA, Psoriatic Arthritis; BCC, Basal cell carcinoma; OR, odds ratio; CI, confidence interval; IVW, inverse variance weighted method. Statistical significance: p < 0.05.

All retained SNPs exhibited F-statistics greater than 10, indicating a strong association between the instrumental variables and PsO and PsA (**Tables S2, S3**). As a result, our study is resilient against weak instrument bias, enhancing the validity of our findings. In our investigation, we conducted a genetic analysis utilizing the summary GWAS database as the exposure and the FinnGen database as the outcome for PsO. The genetically predicted PsO, per log-odds ratio increase, did not exhibit a significant association with the risk of BCC, cSCC, and CM. The odds ratios (with corresponding 95% confidence intervals) for BCC, cSCC, and CM were 1.00 (0.99, 1.01) (P_Ivw_ = 0.990), 0.94(0.89, 1.00) (P_Ivw_ = 0.065), and 0.99 (0.98, 1.01) (P_Ivw_ = 0.239), respectively (**Figure 2**). For PsA, our analysis involved utilizing the FinnGen database as the exposure and the summary GWAS database as the outcome. Univariate regression analysis demonstrated that PsA did not exhibit a significant association with the risk of cSCC and CM, with odds ratios (with corresponding 95% confidence intervals) of 1.00 (1.00, 1.00) (P_Ivw_ = 0.214) and 1.00 (1.00, 1.00) (P_Ivw_ = 0.477), respectively. The MR-Egger, weighted median estimates, and MR-PRESSO also showed the consistent results. PsA did exhibit a significant association with the decrease risk of BCC, with an odds ratio of 0.94(0.90,0.99) (P_Ivw_ = 0.016) (**Figure 3**). However, in our multivariate regression analysis that included these three risk factors, PsA was not found to be associated with BCC, while skin tanning, radiation-related disorders of the skin and subcutaneous tissue, and telomere length remained significantly associated with BCC (P < 0.05) (**Figure 4**). Scatter plot of the primary MR analysis and leave-one-out sensitivity analysis were presented in the **Figures S1** and **S2**.

The heterogeneity test revealed the presence of heterogeneity in the analysis (PsO vs cSCC; PsO vs CM; PsA vs BCC). Therefore, we employed the random-effects model as the main method to address the heterogeneity (26). No significant evidence of horizontal pleiotropy was detected as indicated by the MR-Egger intercept. Steiger filtering did not detect any SNPs with an orientation of “FALSE.” Furthermore, MR PRESSO did not identify any potential outliers. Comprehensive records of these sensitivity analyses are furnished in **Table S4**. Summarized information regarding all candidate IVs is presented in **Tables S5–S10**.

## Discussion

In this study, we comprehensively investigated the relationship between both psoriasis and psoriatic arthritis, and skin tumors. Our findings indicate that PsO and PsA are not causally associated with genetic risk of skin cancer. Although PsA may be associated with BCC, this association is likely confounded by other risk factors underlying the BCC. Our findings may help to guide the clinical management of psoriasis patients with high risk factors for skin tumors. Additionally, these findings contribute to the ongoing exploration of the genetic mechanisms underlying the progression of PsO and PsA towards the development of skin cancer.

There are currently numerous speculations regarding the reasons behind the increased risk of skin cancer associated with PsO and PsA. Presently, there is a prevailing belief that individuals afflicted with conditions such as rheumatoid arthritis (RA), inflammatory bowel disease, PsO, and PsA may encounter an increased occurrence of skin cancer when undergoing treatment with biologics, such as tumor necrosis factor (TNF)-alpha inhibitors. Interestingly, the incidence of Non-Melanoma Skin Cancer in psoriasis patients treated with TNF inhibitors is nearly six times higher compared to RA, underscoring the influence of disease-related factors, such as prior psoriasis phototherapy (27). Another study also proposed that the heightened propensity for sun-seeking behavior among psoriasis patients could be attributed to the beneficial effects of sunlight (28). However, the lesion skin of psoriasis patients exhibits epidermal dysfunction and diminished expression of filaggrin, likely influenced by cytokines such as IL-2. These alterations theoretically give rise to an augmented risk of skin cancer due to sun exposure increases risk of DNA damage (28, 29).

Despite prior investigations demonstrating an elevated risk of skin cancer in individuals with psoriasis (4, 30), the specific determinants contributing to this heightened susceptibility to skin cancer, including the disease itself, behavioral factors, treatment characteristics, or their combined influence, remain unknow. Futhermore, given the constraints posed by the existing observational studies, there is a dearth of robust evidence to substantiate the association between PsA and skin cancer. MR confers the advantage of direct and precise measurement of genetic variation, impervious to external environmental influences and social behaviors, thereby providing a robust and enduring exposure factor. By selecting representative samples without imposing exclusion criteria and random allocation to each observational group, the MR design effectively minimizes the impact of biases.

Our study possesses several notable strengths. Firstly, we employ multivariate analysis correction, a effect approach in Mendelian studies investigating PsO and PsA. This enables us to account for potential confounding factors and enhance the robustness of our findings. Secondly, we leverage the most recent FinnGen database along with the largest available GWAS database for psoriasis, augmenting the feasibility and comprehensiveness of our research. It is worth noting that certain previous studies have demonstrated that in the absence of enough population quantity or adequate multivariate adjustment, the risk of obtaining contradictory outcomes is heightened (31, 32). Several limitations in our study that warrant consideration. Firstly, the utilization of publicly available aggregated data for GWAS analysis introduces potential challenges in ascertaining subject overlap between the conducted MR analyses across the two samples. Secondly, it is crucial to recognize that our study sample predominantly consisted of individuals from a European population, thus limiting the generalizability of our findings to other ethnicities or geographic regions. Thirdly, although this finding should be interpreted with caution due to the restricted sample size and potential influence of unaccounted clinical covariates, it provides evidence suggesting that PsO and PsA may not exert an independent influence as a risk factor for skin cancer. Hence, conducting studies with larger sample sizes is warranted to further investigate the potential causal relationship between these variables.

In summary, although PsO and PsA as diseases may not inherently amplify the skin cancer risk, environmental elements like sun exposure and therapeutic interventions such as biologics and methotrexate can heighten the probability of skin cancer development. Consequently, for patients affected by PsO and PsA, especially those exposed to skin cancer risk factors, the significance of health education and routine self-examinations is greatly accentuated.

## Data availability statement

The original contributions presented in the study are included in the article/**Supplementary Material**. Further inquiries can be directed to the corresponding author.

## Author contributions

NY and YG designed the study. JW and YL analyzed the data and created the figures. All authors critically revised the manuscript. All authors contributed to the article and approved the submitted version.
