# Supplementary material for: Multivariate Mendelian randomization provides no evidence for causal associations among both psoriasis and psoriatic arthritis, and skin cancer
Source: Front Immunol. 2023 Sep 19;14:1252720. doi: 10.3389/fimmu.2023.1252720 (PMC10546308; doi:10.3389/fimmu.2023.1252720)
Supplement: Supplementary file 1 [file DataSheet_1.pdf]

Supplementary Materials

Title: Multivariate Mendelian Randomization provides no evidence for Causal Associations Among Both Psoriasis and Psoriatic Arthritis, and Skin Cancer.

Nianzhou Yu [1,2], Jiayi Wang[3], Yuancheng Liu[1,2], Yeye Guo[1,2, #]

[1] Department of Dermatology, Hunan Engineering Research Center of Skin Health and Disease, Hunan Key Laboratory of Skin Cancer and Psoriasis, Xiangya Hospital, Central South University, Changsha, Hunan, China.

[2] National Clinical Research Center for Geriatric Disorders, Xiangya Hospital, Central South University, Changsha, Hunan 410008, China.

[3]Xiangya School of Medicine, Central South University, Changsha, China.

Yeye Guo is the corresponding author.

Corresponding author:

Yeye Guo, M.D., Ph.D.

Department of Dermatology, Xiangya Hospital of Central South University, 87 Xiangya Road, Kaifu District, Changsha, Hunan Province, China.

E-mail: yeyeguo@csu.edu.cn

Contents

1. Supplemental Figure section

Figure S1. Visualization of the MR analysis of the effect of PsO on skin cancer.

Figure S2. Visualization of the MR analysis of the effect of PsA on skin cancer.

2. Supplemental Table section

Table S1. Detail information of traits involved in this study.

Table S2. F-statistics for all univariable MR analyses.

Table S3. F-statistics for all multivariable MR analyses.

Table S4. Heterogeneity test and horizontal pleiotropy test.

Table S5. Detail SNPs information of PsO trait on BCC.

Table S6. Detail SNPs information of PsO trait on cSCC.

Table S7. Detail SNPs information of PsO trait on CM.

Table S8. Detail SNPs information of PsA trait on BCC.

Table S9. Detail SNPs information of PsA trait on cSCC.

Table S10. Detail SNPs information of PsA trait on CM.

1. Supplemental Figure section

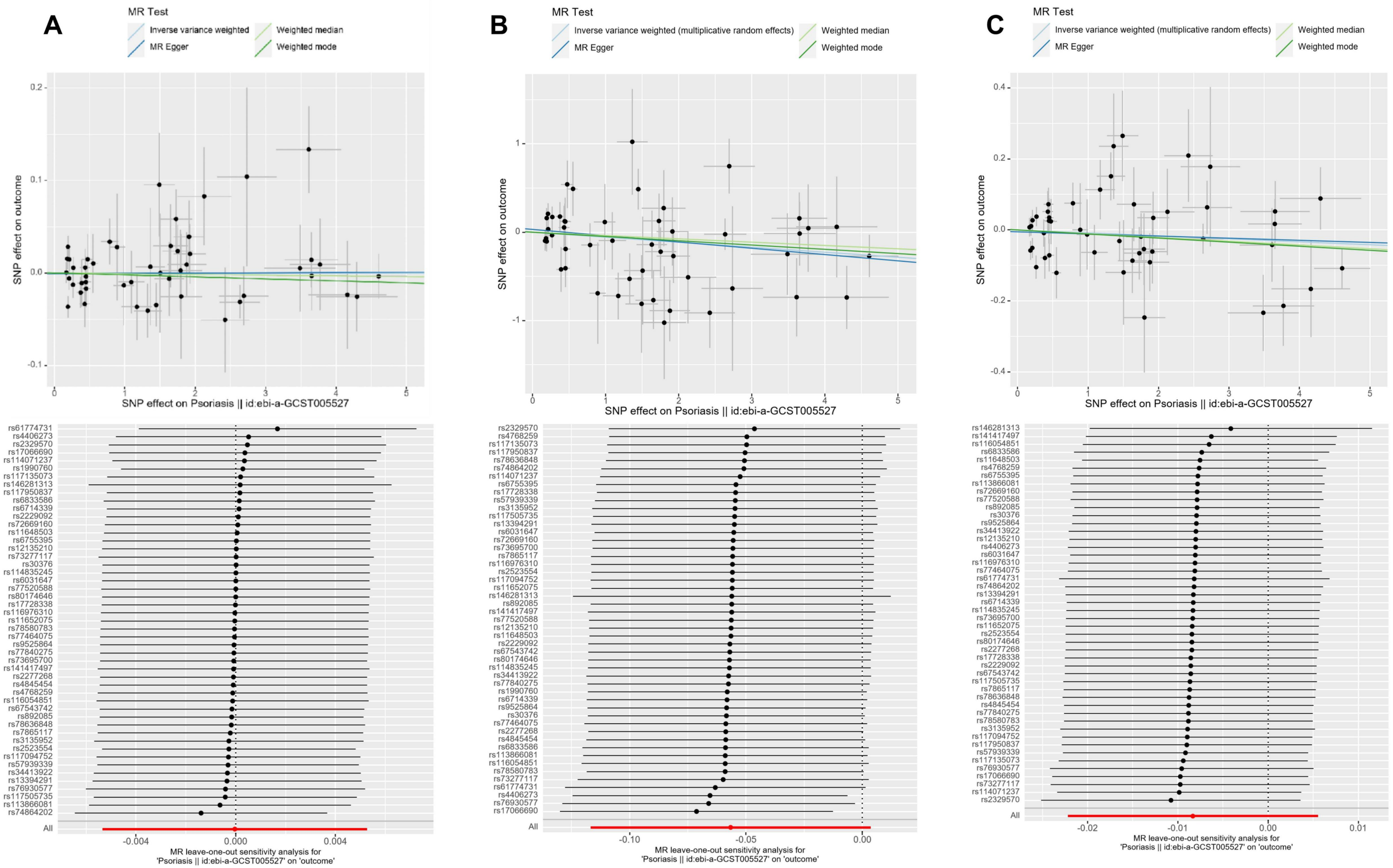

Figure S1. Visualization of the MR analysis of the effect of PsO on skin cancer. (A) Scatter plot of the primary MR analysis and Leave-one-out sensitivity analysis of the effect of PsO on BCC; (B) Scatter plot of the primary MR analysis and Leave-one-out sensitivity analysis of the effect of PsO on cSCC.(C) Scatter plot of the primary MR analysis and Leave-one-out sensitivity analysis of the effect of PsO on CM.

Abbreviations: PsO, Psoriasis; PsA, Psoriatic Arthritis; BCC, Basal cell carcinoma; cSCC, Cutaneous squamous cell carcinoma; CM, CutaneousMelanoma; IVW, inverse-variance weighted; MR, Mendelian randomization.

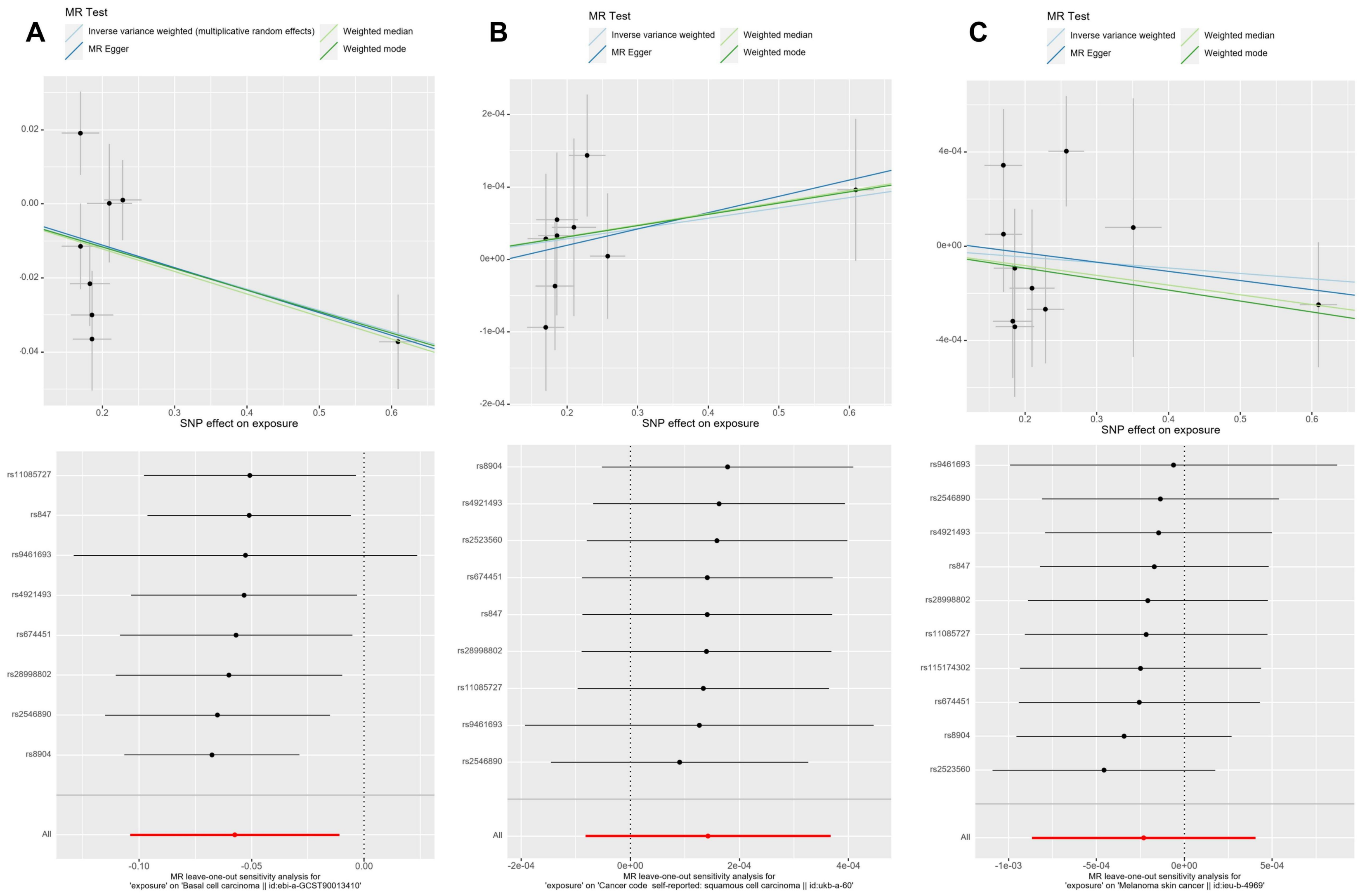

A
MR Test
Inverse variance weighted (multiplicative random effects)
MR Egger
Weighted median
Weighted mode
SNP effect on exposure
rs11085727
rs847
rs9461693
rs4921493
rs674451
rs28998802
rs2546890
rs8904
All
MR leave-one-out sensitivity analysis for
'exposure' on 'Basal cell carcinoma || id:ebi-a-GCST90013410'
B
MR Test
Inverse variance weighted
MR Egger
Weighted median
Weighted mode
SNP effect on exposure
rs8904
rs4921493
rs2523560
rs674451
rs847
rs28998802
rs11085727
rs9461693
rs2546890
All
MR leave-one-out sensitivity analysis for
'exposure' on 'Cancer code self-reported: squamous cell carcinoma || id:ukb-a-60'
C
MR Test
Inverse variance weighted
MR Egger
Weighted median
Weighted mode
SNP effect on exposure
rs9461693
rs2546890
rs4921493
rs847
rs28998802
rs11085727
rs115174302
rs674451
rs8904
rs2523560
All
MR leave-one-out sensitivity analysis for
'exposure' on 'Melanoma skin cancer || id:ieu-b-4969'

Figure S2. Visualization of the MR analysis of the effect of PsA on skin cancer. (A) Scatter plot of the primary MR analysis and Leave-one-out sensitivity analysis of the effect of PsA on BCC; (B) Scatter plot of the primary MR analysis and Leave-one-out sensitivity analysis of the effect of PsA on cSCC.(C) Scatter plot of the primary MR analysis and Leave-one-out sensitivity analysis of the effect of PsA on CM.

Abbreviations: PsA, Psoriatic Arthritis; BCC, Basal cell carcinoma; cSCC, Cutaneous squamous cell carcinoma; CM, CutaneousMelanoma; IVW, inverse-variance weighted; MR, Mendelian randomization.

2. Supplemental Table section

Supplementary Table S1. Detail information of traits involved in this study.

Traits	Study	Ancestry	nCase	Sample size
PsO	Tsoi L et.al[1]	European	10,588	33,394
PsA	FinnGen R9 data	European	3,186	244,048
BCC	Adolphe C et.al[2]	European	10,588	392,871
	FinnGen R9 data		18,982	305,750
cSCC	Neale Lab	European	404	337,159
	FinnGen R9 data	European	3,251	290,408
CM	UK Biobank	European	3,751	375,767
	FinnGen R9 data	European	2993	290130
Ease of skin tanning	MRC-IEU(ID: ukb-b-533)	European	NA	453,065
Radiation-related disorders of the skin	FinnGen	European	5,519	213,273
Telomere length	Codd et.al (ID: ieu-b-4879)	European	NA	472,174

Abbreviation: PsO, Psoriasis; PsA, Psoriatic Arthritis; BCC, Basal cell carcinoma; cSCC, Cutaneous squamous cell carcinoma; CM, Cutaneous Melanoma.

Supplementary Table S2. F-statistics for all univariable MR analyses.

Exposure	PsO			PsA		
Outcome	BCC	cSCC	CM	BCC	cSCC	CM
F-statistics*	167.9	157.1	167.8	111.2	110.7	107.4

*: The reported F-statistics in univariable MR analyses were total F-statistics, and the formula is descripted in the Method. Abbreviation: MR mendelian randomization, PsO, Psoriasis; PsA, Psoriatic Arthritis; BCC, Basal cell carcinoma; cSCC, Cutaneous squamous cell carcinoma; CM, Cutaneous Melanoma.

Supplementary Table S3. F-statistics for all multivariable MR analyses.

Exposure	PsA		
Outcome	BCC		
Adjustment	Ease of skin tanning	Radiation-related disorders of the skin	Telomere length
F_{TS}-statistics*	406.8	86.2	300.1

*: The reported conditional F-statistics (FTS) for PsA were calculated by MVMR package. Abbreviation: MR mendelian randomization, PsA, Psoriatic Arthritis, BCC, Basal cell carcinoma;

Supplementary Table S4. Heterogeneity test and horizontal pleiotropy test.

Exposure	Outcome	Heterogeneity test			Horizontal pleiotropy test		
		Method	Cochran's Q	Q_ pval	Egger_ intercept	Se	pval
PsO	BCC	MR Egger	58.549	0.120	-0.0007	0.004	0.870
		IVW	58.582	0.140			
PsO	cSCC	MR Egger	79.626	0.002	0.0316	0.050	0.536
		IVW	80.282	0.002			
PsO	CM	MR Egger	73.065	0.006	-0.0052	0.012	0.669
		IVW	73.359	0.008			
PsA	BCC	MR Egger	15.835	0.014	0.0010	0.014	0.941
		IVW	15.850	0.026			
PsA	cSCC	MR Egger	4.168	0.760	-2.55E-05	6.70E-05	0.714
		IVW	4.312	0.827			

PsA	CM	MR Egger	10.160	0.253	4.93E-05	0.0002	0.815
		IVW	10.233	0.331			

Abbreviation: PsO, Psoriasis; PsA, Psoriatic Arthritis; BCC, Basal cell carcinoma; cSCC, Cutaneous squamous cell carcinoma; CM, Cutaneous Melanoma.

Table S5. Detail SNPs information of PsO trait on BCC.

SNP	A1	A2	Beta	Se	Pval	Fval
rs113866081	A	G	0.033780489	0.018645393	0.070027337	57.35845516
rs114071237	T	G	-0.030793427	0.02206211	0.162786559	43.93623538
rs114835245	G	A	-0.013579086	0.043644812	0.755703958	44.79300763
rs116054851	C	A	0.002421257	0.013145038	0.853860092	75.83618352

rs11648503	A	G	-0.048146944	0.055606027	0.386567828	37.86517728
rs11652075	T	C	0.002110826	0.067297031	0.974977802	31.27935367
rs116976310	C	A	-0.000126911	0.042585196	0.997622174	34.41464171
rs117094752	C	T	0.042919889	0.031803192	0.17716147	41.46629968
rs117135073	T	C	-0.020900884	0.023430282	0.372368403	49.29696612
rs117505735	A	C	0.038979102	0.024801335	0.116030967	30.52139524
rs117950837	C	A	-0.031347188	0.030539797	0.304685488	30.31483062
rs12135210	T	G	-0.009048555	0.030851916	0.769300573	33.266884
rs13394291	C	T	0.013449001	0.016201428	0.406475429	66.81415512
rs141417497	A	G	0.001455256	0.013486389	0.914070598	47.92730231
rs146281313	C	A	-0.000793	0.005222326	0.879306785	132.3111612
rs17066690	C	A	-0.00923453	0.011958271	0.439978485	58.6393162
rs17728338	A	G	-0.008757695	0.048389655	0.856381074	60.48454624
rs1990760	T	C	-0.190444263	0.0597006	0.001422782	31.54190303
rs2229092	C	A	-0.078243149	0.05829363	0.17952316	32.88284771

rs2277268	A	G	0.031198962	0.058177782	0.591772401	31.02685442
rs2329570	C	T	-0.005969719	0.008752702	0.4952118	55.4033683
rs2523554	T	C	0.147078559	0.060826212	0.015605527	34.7536955
rs30376	T	C	-0.028480118	0.05853198	0.626560519	38.63362758
rs3135952	T	C	0.010664132	0.016592972	0.520425469	71.73529599
rs34413922	T	C	0.020477849	0.020508238	0.318028132	77.94529488
rs4406273	A	G	-0.023982959	0.016328714	0.141897951	1208.958008
rs4768259	A	C	0.004812271	0.018688158	0.796789923	45.98889469
rs4845454	T	C	0.020594452	0.044298094	0.641998733	63.07969313
rs57939339	A	G	0.063975589	0.037640037	0.089193278	44.51769838
rs6031647	G	A	-0.028360042	0.065674274	0.665865858	38.63366146
rs61774731	A	G	-0.011902789	0.00690801	0.084880976	83.50516171
rs6714339	T	C	-0.056966349	0.045001743	0.205559683	40.39713981
rs67543742	G	A	0.069136775	0.059744481	0.247187503	32.87563167
rs6755395	G	A	-0.014138912	0.037401597	0.70540858	37.15793148

rs6833586	G	A	-0.005678576	0.014042202	0.685923359	55.95279491
rs72669160	C	T	-0.03761362	0.04396524	0.392257321	46.61059326
rs73277117	C	A	-0.000865141	0.010197919	0.932392555	54.98329922
rs73695700	A	G	0.031405384	0.064832101	0.628094197	62.04635946
rs74864202	A	C	0.036966212	0.01304334	0.004595404	61.26810865
rs76930577	C	T	0.003862278	0.008272935	0.640602195	92.09841681
rs77464075	T	C	0.001430398	0.024739117	0.953892585	35.56028456
rs77520588	A	G	-0.003869846	0.024398491	0.873976095	60.92076989
rs77840275	C	T	0.013180433	0.046262509	0.775716705	39.65505709
rs78580783	C	A	0.004834142	0.04667036	0.917502222	42.2007555
rs78636848	A	C	0.017627082	0.027310725	0.518650453	45.43167949
rs7865117	T	G	0.038069123	0.035440171	0.282742006	42.02134783
rs80174646	T	G	-0.022236717	0.061234598	0.716500485	34.37441395
rs892085	A	G	0.084081113	0.062944583	0.181616005	39.02536227
rs9525864	G	A	0.018579276	0.057425172	0.746287173	77.2418699

Table S6. Detail SNPs information of PsO trait on cSCC.

SNP	A1	A2	Beta	Se	Fval	Pval
rs113866081	A	G	0.07365396	0.179871039	57.35845516	0.682185946
rs114071237	T	G	-0.398414806	0.217882284	43.93623538	0.067462813
rs114835245	G	A	0.116774284	0.436370102	44.79300763	0.789004329
rs116054851	C	A	0.012049116	0.131170174	75.83618352	0.926810337
rs11648503	A	G	-0.127779772	0.54463621	37.86517728	0.81450764
rs11652075	T	C	-0.572166157	0.657116936	31.27935367	0.383905976
rs116976310	C	A	-0.289341701	0.406395511	34.41464171	0.476482913
rs117094752	C	T	-0.184608394	0.313690577	41.46629968	0.556193547
rs117135073	T	C	-0.376266907	0.163618059	49.29696612	0.021467143

rs117505735	A	C	-0.239801594	0.245270928	30.52139524	0.328222303
rs117950837	C	A	-0.615889103	0.226387063	30.31483062	0.006518057
rs12135210	T	G	-0.087813731	0.294856808	33.266884	0.765841904
rs13394291	C	T	-0.125945745	0.156982867	66.81415512	0.422385369
rs141417497	A	G	-0.070886726	0.132229634	47.92730231	0.591897704
rs146281313	C	A	-0.058529218	0.052063007	132.3111612	0.260928309
rs17066690	C	A	0.277856803	0.11577785	58.6393162	0.016398956
rs17728338	A	G	-0.908531044	0.474051834	60.48454624	0.055298612
rs1990760	T	C	0.832474213	0.587045061	31.54190303	0.156168726
rs2229092	C	A	0.12537361	0.567640627	32.88284771	0.825195269
rs2277268	A	G	1.146889224	0.569794336	31.02685442	0.044134347
rs2329570	C	T	-0.17179415	0.083010408	55.4033683	0.038494549
rs2523554	T	C	-0.540694197	0.595617188	34.7536955	0.363990186
rs30376	T	C	0.995592882	0.571940709	38.63362758	0.081731368
rs3135952	T	C	-0.140201089	0.159125847	71.73529599	0.378279665

rs34413922	T	C	0.004378733	0.201450913	77.94529488	0.982658562
rs4406273	A	G	0.337327482	0.16003613	1208.958008	0.035046494
rs4768259	A	C	-0.473245047	0.184814454	45.98889469	0.010447669
rs4845454	T	C	0.641649257	0.433939218	63.07969313	0.139230781
rs57939339	A	G	-0.543815422	0.370298996	44.51769838	0.14194548
rs6031647	G	A	-1.082297821	0.653393572	38.63366146	0.097635666
rs61774731	A	G	-0.008842046	0.06812561	83.50516171	0.896732305
rs6714339	T	C	0.475495375	0.439723824	40.39713981	0.279541436
rs67543742	G	A	0.160608791	0.590061379	32.87563167	0.785475951
rs6755395	G	A	-0.568719826	0.355334752	37.15793148	0.109483617
rs6833586	G	A	0.014591946	0.137061349	55.95279491	0.915215104
rs72669160	C	T	-0.421206841	0.432505022	46.61059326	0.330117445
rs73277117	C	A	-0.004169196	0.098908721	54.98329922	0.966377561
rs73695700	A	G	-0.777101239	0.6446659	62.04635946	0.228036375
rs74864202	A	C	-0.203454119	0.122546734	61.26810865	0.096870892

rs76930577	C	T	0.043088945	0.080670528	92.09841681	0.593248199
rs77464075	T	C	0.152385149	0.239934514	35.56028456	0.525355752
rs77520588	A	G	-0.085730148	0.235662976	60.92076989	0.716020175
rs77840275	C	T	0.284049339	0.453969123	39.65505709	0.531510493
rs78580783	C	A	0.750698842	0.440103597	42.2007555	0.088057967
rs78636848	A	C	-0.465505359	0.198097257	45.43167949	0.018779317
rs7865117	T	G	-0.232818828	0.341205545	42.02134783	0.495022806
rs80174646	T	G	0.270335781	0.603587645	34.37441395	0.654238671
rs892085	A	G	-0.383096807	0.616367445	39.02536227	0.534244613
rs9525864	G	A	0.888918067	0.55541691	77.2418699	0.109498363

Table S7. Detail SNPs information of PsO trait on CM.

SNP	A1	A2	Beta	Se	Fval	Pval
rs113866081	A	G	-0.037981258	0.042958279	57.35845516	0.376619069

rs114071237	T	G	0.113878934	0.051232769	43.93623538	0.026230956
rs114835245	G	A	-0.013104001	0.100699	44.79300763	0.89646326
rs116054851	C	A	-0.056630773	0.029848155	75.83618352	0.057788933
rs11648503	A	G	-0.395987408	0.125153335	37.86517728	0.001556072
rs11652075	T	C	0.044113866	0.155539846	31.27935367	0.776703307
rs116976310	C	A	-0.079434642	0.097592044	34.41464171	0.415675953
rs117094752	C	T	0.095480214	0.073613245	41.46629968	0.194613257
rs117135073	T	C	0.086320875	0.053890874	49.29696612	0.109206109
rs117505735	A	C	0.023983921	0.057251934	30.52139524	0.675275358
rs117950837	C	A	0.096736625	0.071264964	30.31483062	0.174647183
rs12135210	T	G	-0.057859904	0.071038349	33.266884	0.415365273
rs13394291	C	T	-0.010811136	0.03747687	66.81415512	0.77298324
rs141417497	A	G	-0.066860639	0.030776996	47.92730231	0.029823812
rs146281313	C	A	-0.023446257	0.012133776	132.3111612	0.053320839
rs17066690	C	A	0.023616547	0.027757387	58.6393162	0.394869208

rs17728338	A	G	0.077407054	0.110876402	60.48454624	0.485090756
rs2229092	C	A	0.119424696	0.134869958	32.88284771	0.375897617
rs2277268	A	G	0.050839987	0.134538595	31.02685442	0.705516744
rs2329570	C	T	0.02057738	0.020365778	55.4033683	0.312308439
rs2523554	T	C	0.055375525	0.140518905	34.7536955	0.693522845
rs30376	T	C	-0.242368085	0.13526077	38.63362758	0.073155725
rs3135952	T	C	0.01757089	0.038257458	71.73529599	0.646033019
rs34413922	T	C	-0.031807207	0.047161306	77.94529488	0.500035169
rs4406273	A	G	-0.021618932	0.037723321	1208.958008	0.566582439
rs4768259	A	C	-0.048416908	0.04325133	45.98889469	0.262956035
rs4845454	T	C	0.139615615	0.102377734	63.07969313	0.172652472
rs57939339	A	G	0.17759139	0.085649391	44.51769838	0.038128617
rs6031647	G	A	-0.203388037	0.151752198	38.63366146	0.180159468
rs61774731	A	G	-0.009396553	0.015996082	83.50516171	0.556915999
rs6714339	T	C	-0.023659659	0.104103231	40.39713981	0.820212917

rs67543742	G	A	0.128133006	0.138075872	32.87563167	0.353412844
rs6755395	G	A	-0.136927427	0.085882144	37.15793148	0.110854455
rs6833586	G	A	-0.039926883	0.03251596	55.95279491	0.219478315
rs72669160	C	T	-0.158599825	0.100757355	46.61059326	0.115469675
rs73277117	C	A	0.014254265	0.023469951	54.98329922	0.543624587
rs73695700	A	G	0.000447956	0.14715475	62.04635946	0.997571153
rs74864202	A	C	-0.011667423	0.028718244	61.26810865	0.684542629
rs76930577	C	T	0.004617827	0.019180499	92.09841681	0.809743943
rs77464075	T	C	-0.030057007	0.056513421	35.56028456	0.59482567
rs77520588	A	G	-0.053015305	0.055608134	60.92076989	0.340401011
rs77840275	C	T	0.164893483	0.107068086	39.65505709	0.123540705
rs78580783	C	A	0.17287908	0.108840253	42.2007555	0.112201686
rs78636848	A	C	0.043642766	0.063162963	45.43167949	0.489593805
rs7865117	T	G	0.065049005	0.082009754	42.02134783	0.427669342
rs80174646	T	G	0.05772476	0.142901508	34.37441395	0.686251051

rs892085	A	G	-0.31723824	0.145681666	39.02536227	0.029434886
rs9525864	G	A	-0.21868667	0.131568777	77.2418699	0.09648327

Table S8. Detail SNPs information of PsA trait on BCC.

SNP	A1	A2	Beta	Se	Pval	Fval
rs11085727	T	C	-0.161451374	0.064157089	0.011852658	39.93374183

rs2546890	G	A	0.00453464	0.047478688	0.923910578	77.64299615
rs28998802	A	G	0.000785161	0.076341523	0.991794024	45.11749947
rs4921493	C	T	-0.117855659	0.062237055	0.058270501	44.30902027
rs674451	C	T	-0.067407359	0.068160606	0.322688124	42.47856917
rs847	C	T	-0.196350915	0.075074273	0.008911724	48.37091621
rs8904	A	G	0.112483215	0.066552216	0.090999296	42.12078218
rs9461693	A	G	-0.061090732	0.020939271	0.003528276	550.0642999

Table S9. Detail SNPs information of PsA trait on cSCC.

SNP	A1	A2	Beta	Se	Fval	Pval

rs11085727	T	C	0.00029542	0.000499418	39.93374183	0.554165534
rs2523560	T	C	1.76E-05	0.000336122	106.2187937	0.958267765
rs2546890	G	A	0.000628903	0.00036882	77.64299615	0.088161305
rs28998802	A	G	0.000211707	0.000584377	45.11749947	0.717144005
rs4921493	C	T	-0.000201074	0.000484128	44.30902027	0.677898475
rs674451	C	T	0.000167563	0.000528642	42.47856917	0.751267542
rs847	C	T	0.000176481	0.000590867	48.37091621	0.765183429
rs8904	A	G	-0.000552057	0.000516334	42.12078218	0.284985524
rs9461693	A	G	0.000157772	0.000161012	550.0642999	0.327147356

Table S10. Detail SNPs information of PsA trait on CM.

SNP	A1	A2	Beta	Se	Fval	Pval

rs11085727	T	C	-0.000501689	0.001360911	39.93374183	0.712394359
rs115174302	T	C	0.000225952	0.00156399	78.50760376	0.88512826
rs2523560	T	C	0.001564391	0.000911197	106.2187937	0.086005974
rs2546890	G	A	-0.001170246	0.001004138	77.64299615	0.243847424
rs28998802	A	G	-0.000849378	0.001593007	45.11749947	0.593901046
rs4921493	C	T	-0.001738618	0.001316994	44.30902027	0.186787626
rs674451	C	T	0.000300232	0.001437436	42.47856917	0.834552698
rs847	C	T	-0.001840022	0.00160105	48.37091621	0.250448951
rs8904	A	G	0.002021585	0.001402666	42.12078218	0.149515477
rs9461693	A	G	-0.000407605	0.000434831	550.0642999	0.348559916

Reference

1. Tsoi L, Spain S, Knight J, et al. Identification of 15 new psoriasis susceptibility loci highlights the role of innate immunity. Nature genetics. 2012;44(12):1341-8.
2. Adolphe C, Xue A, Fard A, et al. Genetic and functional interaction network analysis reveals global enrichment of regulatory T cell genes influencing basal cell carcinoma susceptibility. Genome medicine. 2021;13(1):19.
